# Storage and use of residual newborn screening dot blood samples in Italy

**DOI:** 10.1186/1824-7288-37-25

**Published:** 2011-05-21

**Authors:** Carlo Petrini, Antonella Olivieri, Carlo Corbetta, Roberto Cerone, Giuliano D'Agnolo, Adriano Bompiani

**Affiliations:** 1 Bioethics Unit, Office of the President, National Institute of Health (Istituto Superiore di Sanità), Via Giano della Bella 34, I-00162 Rome, Italy; 2 The Italian National Registry of Infants with Congenital Hypothyroidism, Department of Cell Biology and Neurosciences, National Institute of Health (Istituto Superiore di Sanità). Viale Regina Elena 299, I-00161, Rome, Italy; 3 Regional Newborn Screening Center, Children's Hospital "V. Buzzi", A.O. Istituti Clinici di Perfezionamento. Via Castelveltro 32, I-20152 Milan, Italy; 4 University Dept. of Paediatrics, G. Gaslini Institute, Largo Gerolamo Gaslini 5, I-16147 Genoa, Italy. President of the Italian Society for the Study of Hereditary Metabolic Diseases and Neonatal Screening (SIMMESN), Italy; 5 Italian National Committee for Biosecurity, Biotechnology and Life Sciences. Via della Mercede 96, I-00187 Rome, Italy; 6 Honorary President, Italian National Bioethics Committee. Via della Mercede 96, I-0087 Rome, Italy

## 

State newborn screening programs are essential to protect the health of children. Moreover, blood-spot card stocks and, in general, collections of biological specimens have a significant research potential. Children are a vulnerable research population, as they lack the capacity for consenting to their participation. However, unlike members of such other populations, children's vulnerability is temporary and does not arise from a disorder: most children will become healthy adults. Therefore, paediatric biobanks raises unique ethical issues: parental authorization, assent and consent of the child when he will mature the capacity to make independent decisions, degree of communication required with the parents and the child, new use of data and/or samples collected previously, potentially lengthy period of storage, long-term studies, withdrawal from storage and/or research, return of results, and others. Codes, guidelines and policy documents from national (e.g.: National Bioethics Committees), international and supranational (e.g.: OECD, UNESCO, WHO, WMA, European Union, Council of Europe) institutions are available. These documents give important guidance but clear-cut national policies are needed for the inclusion of children's biological specimens in population biobanks: lack of openness may lead to the loss of public trust in research.

In Italy neonatal screening program has been mandatory for congenital hypothyroidism, cystic fibrosis, and phenylketonuria since 1992. Moreover, on the basis of regional laws other screening programs for endocrinopathies and inborn errors of metabolism are performed in some Italian Regions. This implies that every year about 550,000 dried blood spot cards from all Italian newborns are collected and analyzed in the 32 Screening Centers active in our country.

After newborn screening has been completed many screening laboratories retain the residual dried blood samples (DBS) for various purposes, including program evaluation, quality assurance, and biomedical research. In 2006 the Italian Society for the Study of Metabolic Diseases and Neonatal Screening (SIMMESN) recommended a storage of at least 5 years protecting privacy of newborns and their families. However, a more recent survey performed by the SIMMESN revealed a significant dishomogeneity among screening laboratories in the management of residual DBS in terms of time of retention, requirement of parental consent, and use of the samples.

Because of this huge variability and to achieve an appropriate balance between respecting the rights of newborns and their parents versus the potential for public good deriving from the use of DBS for research purposes, the Joint Group National Committee of Bioethics - National Committee for Biosafety Biotechnologies and Life Sciences have recommended developing a National Network of Regional Newborn Screening Repositories for collection of residual DBS, coordinated by the Italian National Institute of Health [1].

In particular, the Joint Group recommends:

- to overcome some current organisational problems of neonatal screening (number of screened babies per screening center, cut-off values, recall index, analytical strategies of selection, efficiency index, etc.);

- to reconsider the regulations for the compulsory short-term/medium-term preservation of the samples, in the newborn's medical - legal interest;

- to establish long-term collections of residual material from compulsory neonatal screening;

- to safeguard, with adequate regulations, the current collections present in some neonatal screening Centres to avoid their deterioration or destruction;

- to promote the training of the staff operating in the various Centres, with regards to not only the "technical" care of the samples (correct collection, correct use, appropriate preservation, etc.), but also of the rights of the newborn, child and family, inherent to appropriate information, confidentiality, personal data protection and privacy.

The establishment of a national network of regional repositories implies adopting uniform criteria for long-term retention and for an effective education for parents about DBS and control over their retention and use. The latter point is relevant because the maintenance of public trust in such an initiative is crucial and can be obtained only by making public aware of the potential for public health deriving from the use of residual DBS for research purposes.

## Competing interests

The authors declare that they have no competing interests.

## Authors' contributions

*Each author was member of the Working Group that published the document "Long-term storage of residual dot blot spots from neonatal screening"*.

*Each author has equally contributed to the manuscript*.

*All authors read and approved the final manuscript*.
